# Embodied musical experiences in the metaverse: a review of sensorimotor integration in VR-based music performance

**DOI:** 10.3389/fpsyg.2026.1826915

**Published:** 2026-09-30

**Authors:** Yuyi Sun, Xinyue Li, Xuefeng Wei

**Affiliations:** College of Education, Ludong University, Yantai, China

**Keywords:** cross-modal compensation, embodied cognition, music performance, predictive processing, sensorimotor integration, virtual reality

## Abstract

Virtual reality and the musical metaverse decompose traditional music performance into isolated visual, auditory, and tracking modules. While these immersive environments provide strong audiovisual feedback, they severely weaken the tactile and proprioceptive cues necessary for fine motor control. This review critically synthesizes empirical evidence from the past 5 years to evaluate whether audiovisual feedback can effectively compensate for the absence of haptic feedback. We analyze this cross-modal compensation through the lens of predictive processing and sensorimotor integration. Current data suggest that audiovisual feedback can support short-term spatial orientation and rhythmic accuracy in macro-motor tasks, such as percussion. However, evidence for effective compensation becomes substantially weaker in high-complexity tasks requiring fine motor control, such as piano and bowed string instruments. In these scenarios, physical resistance and haptic feedback appear difficult to replace. Furthermore, continuous temporal micro-delays in VR can create persistent action–feedback mismatches. These mismatches may increase monitoring demands and disrupt psychological flow. Whether they also exacerbate music performance anxiety remains an untested hypothesis. Pedagogically, XR technologies facilitate basic concept visualization, but they currently lack robust evidence for durable fine-motor procedural learning and real-instrument transfer. We conclude that the existing literature often conflates short-term usability gains with long-term skill transfer due to methodological limitations. Future research should adopt longitudinal designs, include real-instrument baselines, and treat haptic feedback as a core variable to verify authentic pedagogical transferability.

## 1 Introduction

Music performance in the metaverse dismantles the tightly coupled physical and acoustic feedback of traditional instruments into isolated modules. These modules include visual rendering, motion tracking, and sound synthesis. This heterogeneity poses methodological challenges for theoretical integration (Tuominen and Saarni, 2024; Turchet, 2023). This background reveals a core paradox. VR environments offer strong audiovisual immersion, but they simultaneously weaken the tactile and proprioceptive cues that are essential for fine motor control. Studies show that incorporating kinesthetic and haptic feedback can contribute to realism and perceived control (Fu et al., 2025; Schwartz et al., 2025). Therefore, the critical question is not whether VR is immersive enough. Rather, we must determine to what extent audiovisual feedback can replace the sense of agency and expressive precision that touch naturally supports.

To address this, we define the core mechanisms within a computational neuroscience framework. We specifically utilize the concepts of prediction error minimization and generative models. Research confirms that bodily movement is not a peripheral variable; it is an integral part of predictive processing itself (Ishida and Nittono, 2025). Based on this foundation, we operationalize sensorimotor integration into three sets of measurable metrics. The first set involves temporal coupling, which includes latency and audiovisual synchronization errors (Tsioutas and Xylomenos, 2023; Tsioutas et al., 2025; Turchet, 2023). The second set measures performance output, such as rhythmic accuracy. The third set consists of kinematic indicators, such as motion trajectories. These objective metrics provide more direct indicators of motor and sensorimotor performance than subjective questionnaires, while subjective measures remain important for constructs such as presence, agency, and flow (Yu et al., 2023; Boem et al., 2025).

This view is consistent with broader evidence from embodied music learning. Action observation, auditory–motor imagery, and visuo-motor or audio-motor training studies show that musical learning depends on tightly coupled perception–action mappings rather than on visual information alone. For example, action observation training with egocentric views of a pianist’s hand and congruent auditory feedback has been shown to facilitate the acquisition of key sequences, dynamics, and timing in novice learners (Paolini et al., 2024). Similarly, auditory and motor imagery abilities modulate melodic learning and memory (Brown et al., 2013), and different visuo-motor versus audio-motor piano-training regimes produce different patterns of cross-modal transfer (Engel et al., 2012). These findings support the idea that XR systems should be evaluated not only by their visual realism or presence, but also by whether they preserve action–sound–touch contingencies relevant to instrumental skill acquisition. This concern is consistent with the broader motor-learning principle that learning and transfer depend partly on the correspondence between sensory information available during practice and that available during later performance.

In summary, this paper critically synthesizes recent empirical research most directly relevant to VR/XR music performance and sensorimotor compensation, with priority given to studies published within approximately the past 5 years. We aim to strictly distinguish between compensating for subjective presence and compensating for performance control precision. Multimodal feedback and visual cues can improve certain user experiences (Tai et al., 2024; Yu et al., 2023). However, the existing literature is widely limited by small sample sizes and short-term tasks, which restricts robust causal inferences. Therefore, this review goes beyond asking whether VR music is practically feasible. It primarily explores under what conditions and through which mechanisms VR can truly approximate a genuine embodied musical experience (Tai et al., 2024; Tuominen and Saarni, 2024).

### 1.1 Review scope and selection strategy

This mini review adopts a narrative, concept-driven synthesis rather than a formal systematic review. To avoid conflating direct evidence with broader mechanistic evidence, we distinguish between two levels of literature. The first comprises direct empirical studies of active music performance or instrumental learning in immersive VR, AR, or MR environments, particularly studies reporting timing, kinematic, haptic, embodiment, motor-control, or transfer-related outcomes. Recent studies published within approximately the past 5 years were prioritized for this core evidence base. The second level comprises adjacent mechanistic evidence relevant to interpreting the limited music-XR literature, including haptic and force-feedback research on digital musical instruments, VR motor learning and real-world skill transfer in rehabilitation, sport, and surgical simulation, specificity-of-practice research in motor learning, delayed auditory-feedback studies in music performance, and experimental work on sense of agency.

Searches covered Web of Science, Scopus, PubMed, Google Scholar, IEEE Xplore, ACM Digital Library, major publisher databases, and relevant proceedings of the International Conference on New Interfaces for Musical Expression (NIME). Search terms combined “virtual reality,” “mixed reality,” “extended reality,” “augmented reality,” “music performance,” “musical instrument learning,” “digital musical instrument,” “haptic feedback,” “force feedback,” “sensorimotor integration,” “motor learning,” “skill transfer,” “embodiment,” “presence,” “agency,” “delayed auditory feedback,” “sensorimotor synchronization,” and “latency.”

The relatively small direct music-XR evidence base reflects the narrow intersection of these criteria rather than the size of the broader immersive-music literature. Many studies concern passive music listening, immersive concerts, rehabilitation without active instrumental performance, system demonstrations or usability evaluations without motor-performance outcomes, or non-immersive digital musical interfaces. Such studies were not treated as direct evidence that audiovisual feedback can compensate for reduced haptic information during instrumental learning. Adjacent-domain studies were therefore used to clarify mechanisms and boundary conditions, but not as substitutes for direct evidence of music-performance transfer. Because the aim remained theoretical integration rather than exhaustive evidence mapping, no formal PRISMA screening procedure was conducted.

## 2 Empirical evidence of cross-modal compensation

### 2.1 Behavioral and kinematic outcomes

Current data primarily indicate that cross-modal compensation is more likely to succeed in percussion and rhythm-based tasks. Pinkl et al. (2024) conducted a mixed reality drumming training study with 20 beginners. They found that the mixed reality group showed an additional 239 ms improvement in absolute timing error across four exercises compared to the video practice group (*z* = 3.520, *p* < 0.001). The authors noted that this difference roughly means the timing error reduction in the mixed reality group was about twice that of the video group. Tai et al. (2024) investigated five practice sessions with Jazz-Drum-VR over 4 weeks. Their study showed that the system-recorded accuracy increased from 21.63% (SD = 14.34) in the first session to 50.09% (SD = 14.34) in the fifth session. This suggests that audiovisual synchronization can indeed support considerable behavioral gains in tasks that rely on large muscle groups, macro-spatial orientation, and beat repetition. However, this evidence is mostly limited to rhythmic exercises of low to medium complexity. It cannot be directly generalized to high-speed, polyphonic, or highly fine motor control tasks.

Evidence from keyboard and bowed string instruments suggests a different trend. Evidence for pure audiovisual compensation becomes more limited when tasks rely heavily on fingertip contact, key resistance, bow pressure, and continuous micro-adjustments. Banquiero et al. (2024) investigated mixed reality piano learning with 33 participants. They found that the median teacher rating under the mixed reality condition was 8.75 (IQR = 1.75). This was significantly higher than the 7.38 (IQR = 2.63) rating under the Synthesia condition (*U* = 197.5, *Z* = 2.23, *p* = 0.027, *r* = 0.389). However, this study retained a physical piano keyboard. Therefore, the findings show that visual overlays combined with a physical keyboard produced better performance ratings than the flat-screen comparison condition. It does not prove that a haptic-free virtual keyboard is sufficient. More direct evidence comes from Fu et al. (2025). They added kinesthetic and vibration gloves to a purely virtual piano. The 14 participants scored significantly higher on clarity, realism, enjoyment, and satisfaction compared to the no-feedback and vibration-only conditions. These findings suggest that kinesthetic and vibrotactile feedback may improve perceived realism and control in virtual keystroke tasks. However, given the small sample and largely subjective outcomes, they do not establish haptic feedback as a universally necessary condition.

Studies on bowed string instruments also point in the same direction. Campo et al. (2023) asked 11 amateur violin learners to perform 2D and 3D lead-avatar imitation training using HoloLens 2. They used motion capture to compare gesture resemblance, smoothness, and presence. The results showed that the 3D condition yielded higher immediate performance and presence. However, the learning effect only appeared in the 2D condition. This implies that stronger stereoscopic visual cues can improve immediate imitation. They do not automatically translate into more stable skill acquisition. Based on the current literature, successful compensation in percussion is primarily reflected in reduced timing errors. Conversely, the apparent limits of compensation in keyboard and string-instrument studies manifest differently. In the available studies, reduced physical haptics is often accompanied by alternative kinesthetic feedback, retained physical instruments, or simplified task demands.

### 2.2 Neurophysiological correlates and methodological weaknesses

Neurophysiological evidence currently remains relatively weak. Weiss et al. (2025) conducted an EEG study with 32 participants. They observed separable electrophysiological markers in the centro-parietal and frontal regions between detected and undetected pseudo-haptic manipulations. This indicates that cross-modal pseudo-haptics are not merely subjective illusions but possess measurable neural correlates. The limitation is that this task employs a general VR haptic illusion paradigm rather than a music performance paradigm. Therefore, it indicates neural sensitivity to audiovisual-induced pseudo-haptic manipulations, rather than demonstrating functional replacement of tactile feedback during music performance. It does not prove that audiovisual feedback fully compensates for the lack of tactile feedback during music performance.

Music-related studies have similarly not yet bridged this gap. Liang et al. (2025) conducted an fNIRS study with 50 participants. They reported that VR music stimulation more strongly enhanced functional connectivity in the bilateral primary somatosensory cortex, premotor area, and supplementary motor area compared to VR motor observation and motor imagery. However, their task essentially compared VR music stimulation, observation, and imagery. It did not directly contrast a haptic-free VR instrument with a real physical instrument. Hopkins et al. (2025) further evaluated the BrAIn Jam system. They showed that a random forest classifier achieved accuracy rates of 0.932 and 0.949 for the data of two drummers. They also observed significant differences in the left temporoparietal junction. However, this study involved only two participants. The authors explicitly cautioned that researchers must interpret these results within a framework of small sample sizes and high data volumes. Furthermore, Arlati et al. (2025) reviewed 41 VR-EEG embodiment studies. They noted that the existing literature is highly heterogeneous regarding elicitation paradigms, EEG acquisition, preprocessing, and subjective scales. Consequently, no stable EEG biomarkers currently exist to serve as a unified indicator for embodiment. Overall, current neural evidence should be interpreted as showing that sensorimotor networks are sensitive to multisensory mismatches, pseudo-haptic illusions, and altered action–feedback contingencies in VR. It does not yet demonstrate successful cross-modal compensation in the stronger sense of neural replacement of tactile or proprioceptive feedback during music performance.

The small direct music-XR literature should also be interpreted against a broader body of work on haptic musical interfaces and virtual motor learning. Research on digital musical instruments has long treated vibrotactile and force feedback as functionally relevant components of performer–instrument interaction rather than as purely aesthetic additions (Marshall and Wanderley, 2006; Papetti et al., 2023). More recent NIME systems have similarly explored active force feedback for bowing-, plucking-, and continuous-control gestures (Lane-Smith et al., 2023). Importantly, this adjacent literature does not show that haptics are uniformly necessary for all musical actions; rather, their contribution varies with interface structure, performer experience, and the control demands of the task (Papetti et al., 2023). Evidence from VR motor learning outside music leads to a similarly conditional conclusion. Reviews in rehabilitation and sport emphasize that improvement inside a virtual environment cannot itself establish transfer to the corresponding real-world task (Levac et al., 2019; Michalski et al., 2019), while evidence from surgical simulation suggests that virtual haptic systems can support learning but may still fail to reproduce the full sensory information available in physical practice (Tan et al., 2025). These adjacent findings strengthen the rationale for examining sensory-motor correspondence and transfer in music, while remaining distinct from direct evidence about instrumental performance.

To make the evidentiary boundaries clearer, Table 1 summarizes the main empirical studies reviewed in this section, including their task type, sample size, design features, haptic or physical-instrument conditions, key outcomes, and major interpretive limitations.

**TABLE 1:** Main empirical studies on VR/XR music performance and sensorimotor compensation.

References	Technology/task	Sample	Design	Haptic or physical baseline	Main outcomes	Evidence boundary
Pinkl et al., 2024	Mixed-reality drumming training	*N* = 20	Comparative training study	No real-instrument transfer test	Larger reduction in timing error than video practice	Supports short-term rhythm training, not long-term transfer
Tai et al., 2024	Jazz-Drum-VR	67 usable cases	Five sessions over 4 weeks	No real-drum baseline	Accuracy increased across sessions; flow and learning value examined	Shows practice-related improvement in VR, but transfer remains unclear
Banquiero et al., 2024	Mixed-reality piano learning	*N* = 33	MR vs. synthesia comparison	Physical keyboard retained	Higher teacher rating in MR condition	Supports visual overlay on real keys, not haptic-free piano learning
Fu et al., 2025	Virtual piano with haptic glove	*N* = 14	Feedback-condition comparison	Kinesthetic/vibration manipulation	Higher clarity, realism, enjoyment, and satisfaction with haptics	Suggests haptics are important for virtual keystroke control
Campo et al., 2023	AR violin imitation	*N* = 11	2D vs. 3D lead-avatar training	Physical violin/imitation task	3D improved presence and immediate performance; learning effect stronger in 2D	Presence and learning may dissociate
Amm et al., 2024	Piano Theory Hub	*N* = 12	Short practice evaluation	No teacher or conventional self-study baseline	Improved correct keystrokes/chords and confidence	Supports short-term concept visualization
Yu et al., 2023	VR dulcimer learning	*N* = 112	Randomized comparison of embodiment and visual cues	Virtual instrument task	Immediate performance and cognitive-load effects	Stronger sample, but still mainly immediate VR performance
Deja et al., 2025	AR piano-roll improvisation	*N* = 6	Four-days controlled prototype study	Limited baseline	Trends in rhythm, flow, musicality; some effects nonsignificant after correction	Promising but underpowered
Hopkins et al., 2025	BrAIn Jam/AI virtual musician	*N* = 2	Neuroadaptive prototype	No generalizable baseline	High classifier accuracy within two drummers	Neural/prototype evidence, very small sample
Liang et al., 2025	VR music stimulation/fNIRS	*N* = 50	VR music vs. observation/imagery	Not a direct instrument-transfer design	Sensorimotor connectivity differences	Neural sensitivity, not haptic replacement
Weiss et al., 2025	Pseudo-haptic VR illusion/EEG	*N* = 32	EEG study of detected vs. undetected pseudo-haptic manipulations	General VR haptic-illusion paradigm	Separable electrophysiological markers were observed	Shows neural correlates of pseudo-haptics, but not music-performance compensation

MR, mixed reality; AR, augmented reality; VR, virtual reality; XR, extended reality; EEG, electroencephalography; fNIRS, functional near-infrared spectroscopy. “Evidence boundary” refers to the main limitation that prevents each study from being interpreted as direct evidence for long-term musical skill transfer or full haptic/proprioceptive compensation.

Table 1 is intentionally restricted to the direct music-related XR evidence discussed in the review. Studies from digital musical instrument research, general motor learning, sport, rehabilitation, surgery, and sense-of-agency research are used as adjacent mechanistic evidence and are therefore synthesized narratively rather than included as direct music-XR studies.

## 3 Psychometric outcomes: agency, presence, and psychological flow

Standardized scales consistently indicate that high-fidelity XR effectively enhances spatial and social presence (Campo et al., 2023; Amm et al., 2024). However, this short-term advantage primarily manifests as a heightened sense of “being there” rather than directly translating into superior learning outcomes.

In contrast, evidence supporting an enhanced sense of agency remains noticeably weak. Future studies could operationalize agency more directly by drawing on standardized or semi-standardized instruments. For example, the Sense of Agency Scale distinguishes positive and negative dimensions of consciously perceived control over one’s mind, body, and the immediate environment (Tapal et al., 2017), whereas embodiment-oriented VR questionnaires often include agency or motor-control subscales alongside body ownership and self-location (Gonzalez-Franco and Peck, 2018). These tools would allow researchers to distinguish between perceived control over action, ownership of the virtual body or instrument, and the broader feeling of spatial presence. At present, however, many XR music studies still rely on indirect proxies such as confidence, perceived naturalness, enjoyment, usability, or self-rated task mastery, rather than direct measures of agency. Consequently, while XR may boost subjective task mastery, it has not yet been stably quantified as true agency. Sense of agency should also be distinguished at the level of measurement. The broader agency literature differentiates explicit judgments of control from more implicit sensorimotor indices, among which intentional binding–the perceived temporal compression between a voluntary action and its sensory consequence–is one of the most widely studied (Moore and Obhi, 2012). This distinction is particularly relevant to XR music systems because a performer may explicitly report high control or naturalness while remaining sensitive at a pre-reflective sensorimotor level to action–feedback delays or mismatches. Future XR music studies could therefore combine explicit agency questionnaires with timing-sensitive implicit measures rather than treating usability, confidence, or presence as interchangeable with agency.

Results regarding psychological flow are highly inconsistent. Flow typically emerges in contexts with simple structures, strong rhythms, and immediate feedback, where low-friction action loops are easily formed (Tai et al., 2024). Conversely, complex tasks often couple strong immersion with poor usability and elevated cognitive load, indicating that presence and flow can dissociate (Boem et al., 2025; Van Damme et al., 2024).

These inconsistencies can be interpreted through a predictive-processing framework. In haptic-reduced XR, tracking delays or drift may create persistent discrepancies between predicted and observed sensory consequences. Unlike physical instruments, where contact and resistance provide additional information for online correction, XR systems may require performers to rely more heavily on visual and auditory monitoring when these cues are reduced or temporally unstable. Such additional monitoring may increase cognitive load, weaken perceived control, and interfere with flow. Whether these sensorimotor conditions also contribute to music performance anxiety in XR remains unknown, because no direct empirical study identified in the present review has tested this relationship.

## 4 Pedagogical transferability: empirical realities vs. speculative claims

Regarding pedagogical transferability, current evidence primarily supports narrow, short-term transfer rather than a generalized upgrade in overall performance ability. This transfer mainly involves spatial orientation, especially in drumming tasks, and the visual learning of basic music theory and key mappings. First-person spatialized cues can significantly accelerate the action-sound-space mapping process for beginners. For example, mixed reality scaffolding in percussion training can improve timing errors by more than twice the amount observed in traditional video practice (Pinkl et al., 2024). Simultaneously, spatial hand-tracking technology can effectively correct nearly 20% of spatial misjudgments (Banquiero et al., 2025). In other words, VR and MR provide positive data regarding basic spatial targeting. However, this differs significantly from the formation of a stable instrumental motor schema.

The current evidence therefore needs to be separated into two levels. At the supported level, XR systems show benefits for short-term spatial orientation, basic concept visualization, engagement, confidence, and immediate timing or accuracy improvements in constrained tasks. At the speculative level, however, claims about durable procedural learning, robust real-instrument transfer, or the replacement of conventional embodied teaching remain insufficiently tested. Without delayed post-tests and real-instrument transfer assessments, extrapolating from immediate performance gains to stable motor learning constitutes an evidentiary leap.

Evidence concerning music theory and visual scaffolding similarly leans toward short-term, foundational transfer. Amm et al. (2024) evaluated the Piano Theory Hub system. They showed that after a 10-min practice session, six beginners increased their correct keystrokes from 3.667/20 to 18.667/20, and their confidence scores rose from 1.167 to 4.5. Six intermediate learners also improved their correct chord count from 4.5 to 15.833. However, the authors explicitly noted the limitations of this study. The research lacked direct comparisons with other self-study methods, and it could not claim superiority over traditional teachers. Furthermore, continuous tracking of learning progress remains necessary. Therefore, these results at most support the conclusion that VR and MR facilitate the visualization of basic concepts and enhance short-term accuracy. They do not sufficiently prove that these technologies can reliably replace conventional teaching pathways. More broadly, recent research on E-STEM inquiry and interdisciplinary learning has highlighted the value of structured, activity-based pedagogical frameworks for integrating technology, disciplinary knowledge, and competency development (Wei and Kong, 2025; Wei et al., 2024).

Related evidence from non-XR music pedagogy further cautions against treating visual guidance as sufficient for durable musical learning. Action observation training can support the acquisition of new piano-related skills when the observed movement is egocentric, instrument-specific, and paired with congruent sound (Paolini et al., 2024). However, such evidence also implies that the effectiveness of visual guidance depends on its coupling with real action–sound contingencies. Likewise, imagery and cross-modal piano-learning studies indicate that auditory, motor, and visual codes interact during melodic learning and memory (Brown et al., 2013; Engel et al., 2012). These findings reinforce the present review’s central argument: XR pedagogy is most likely to support durable learning when it preserves embodied action–perception coupling, rather than when it replaces physical resistance with visual cues alone.

A clear distinction must be made regarding speculative claims about the construction of durable fine-motor procedural learning. Recent studies with stronger designs remain limited in scope. Yu et al. (2023) randomly assigned 112 participants to compare the effects of embodied design and visual cues on immediate virtual dulcimer performance and cognitive load. Tai et al. (2024) employed a single-group time-series design involving five practice sessions over 4 weeks. They found that accuracy increased from 21.63% to 50.09%. Deja et al. (2025) conducted a 4-days controlled study on ImproVisAR with 6 participants. They reported that the AR group showed a trend toward higher expert ratings in rhythm, flow, and musicality. However, multiple results lost statistical significance after corrections for multiple comparisons. Thus, the existing literature supports the idea that XR technologies can promote short-term performance, engagement, and the acquisition of local strategies. The evidence falls far short of proving that these technologies can stably durable instrument-specific motor learning for piano keystrokes, bow pressure control, or complex fingering sequences, especially without long-term controlled trials and transfer tests on real instruments. Directly extrapolating short-term performance improvements to the formation of long-term procedural skills remains an evidentiary leap.

A further unresolved concern is the possibility of negative transfer, which has rarely been examined systematically in the current literature. Banquiero et al. (2025) demonstrated that relying solely on Quest 3 hand tracking results in a total photon-to-hand latency of approximately 70 ms. The average absolute jitter was 13.27 ms, with 95% of events falling within a range of ±35.46 ms and a maximum deviation of 46.12 ms. The authors explicitly stated that such temporal fluctuations are sufficient to disrupt rhythmic stability. Existing research has not answered a critical question. If learners practice extensively in an environment characterized by a lack of physical resistance and the presence of temporal jitter, does their motor system develop a transferable optimization? Alternatively, it may hypothetically encourage adaptive biases toward low-resistance interfaces, altered timing tolerances, or visually guided “air playing,” but this possibility remains to be tested through longitudinal transfer designs. Pinkl et al. (2024) also frankly acknowledged that longitudinal studies are necessary to verify whether their observed benefits can persist. Therefore, a more conservative conclusion is appropriate. VR and MR demonstrate practical value for spatial familiarization, concept visualization, and basic rhythm training. However, the possibility of maladaptive or context-specific motor transfer remains largely unexplored and requires direct empirical testing.

The broader motor-learning literature provides a theoretical reason to take this possibility seriously without treating negative transfer as an established effect. According to the specificity-of-practice account, motor learning can become tuned to the sensory information available during acquisition, such that changing or withdrawing previously available sensory cues can impair subsequent performance (Toussaint et al., 2017). Related reviews of virtual motor training likewise emphasize that transfer depends partly on the correspondence between the perceptual and motor constraints of the training and target environments (Levac et al., 2019; Michalski et al., 2019). Music-specific evidence also shows that temporally altered auditory feedback can modify the timing organization of skilled performance (Pfordresher and Palmer, 2002). Together, these findings do not demonstrate that XR practice causes maladaptive musical learning. They instead identify a plausible, testable mechanism: extensive practice under systematically different visual, auditory, proprioceptive, or resistance conditions may produce context-specific adaptations that do not fully generalize when the learner returns to a physical instrument. Direct tests would require both XR acquisition and subsequent real-instrument retention or transfer phases.

Thus, immediate gains in virtual or augmented environments should be treated as usability or near-transfer outcomes unless they are followed by delayed retention tests and transfer tests on physical instruments.

## 5 Conclusion and recommendations for future empirical designs

Within the limited direct music-XR evidence currently available, cross-modal compensation appears to be task-dependent rather than universally effective. Audiovisual feedback has produced measurable improvements in several studies of rhythm, spatial orientation, and constrained instrumental tasks, whereas evidence is substantially less developed for fine, continuous motor control and long-term transfer to physical instruments. The broader literature on digital musical haptics and virtual motor learning is consistent with this distinction, but it should be treated as mechanistic support rather than direct evidence that the same effects occur in musical XR. For example, mixed reality drumming training resulted in an additional 239 ms improvement in timing error compared to video practice. Meanwhile, the accuracy of Jazz-Drum-VR increased from 21.63% to 50.09%. However, direct evidence becomes substantially more limited once tasks involve fine or continuously regulated motor control, such as piano, violin, or improvisation. Existing studies mainly demonstrate short-term benefits of MR scaffolding with retained physical keys or AR assistance. They struggle to prove that pure audiovisual feedback sufficiently replaces the role of haptics, proprioception, and physical resistance in micro-motor control (Pinkl et al., 2024; Tai et al., 2024; Fu et al., 2025).

Therefore, the safest current judgment is not that VR can already reconstruct embodied music performance. Instead, current evidence most consistently supports its use for short-term spatial familiarization, rhythm training, and visual guidance. It has not yet sufficiently proven its ability to shape fine muscle memory over the long term. More crucially, substantial methodological limitations remain. Sample sizes commonly fall in the *N* = 6, 11, 14, 20, or 33 range. Research designs are mostly short-term, single post-test, quasi-experimental, or small-sample prototype validations. Furthermore, embodiment and agency studies suffer from heterogeneous indicators and inconsistent scales. This evidentiary structure is insufficient to support strong claims regarding long-term transfer, procedural skill construction, or even the replacement of real instrument training. At this stage, the primary risk is not that VR is completely ineffective. Rather, it is the premature extrapolation of short-term usability gains into long-term skill transfer conclusions (Arlati et al., 2025; Fu et al., 2025).

The present evidence base also has a cultural and instrumental limitation. Most direct studies reviewed here concern Western instruments or interaction structures derived from piano, violin, and drum performance. The proposed distinction between macro-motor and fine-motor compensation should therefore not be treated as universally instrument-independent. Fretless, microtonal, and continuously controlled performance traditions–for example, those involving erhu, sarangi, oud, or other instruments in which pitch and timbre depend on continuous finger, pressure, or contact adjustments–may place different demands on tactile and proprioceptive information. Comparative studies across instrumental traditions are needed before broad conclusions about haptic compensation can be generalized beyond the currently narrow sample of instruments. The current evidence nevertheless suggests four provisional design principles for XR music systems. First, visual guidance should be used primarily where spatial targeting, sequencing, or rhythmic mapping is the principal learning problem, rather than being assumed to substitute for all forms of physical feedback. Second, systems intended for fine or continuously regulated motor control should preserve or simulate task-relevant resistance, contact, or kinesthetic information wherever feasible. Third, end-to-end action–visual and action–sound latency and jitter should be measured and reported explicitly, because average frame rate or subjective presence alone cannot characterize sensorimotor fidelity. Fourth, pedagogical systems should be designed for transfer rather than only for in-system performance: visual scaffolds should be progressively faded, and evaluation should include delayed retention and transition to the physical instrument. These principles remain provisional because direct comparative evidence is still limited.

Based on these evidentiary boundaries, future research would benefit from three stronger design priorities. First, researchers should abandon simple single post-test designs and shift toward longitudinal designs with adequate statistical power. Given the prevalence of small samples in this field, adequately powered longitudinal studies are needed. Future studies should justify sample sizes through prospective power analysis based on the expected effect size, study design, and planned analyses. Studies should also include delayed post-tests to examine retention rates rather than just immediate performance. Second, studies must establish a real physical instrument baseline control group. Otherwise, it is impossible to determine whether performance improvements stem from the XR scaffolding itself or merely from additional visual cues. Third, researchers should treat haptic or kinesthetic feedback as an experimentally manipulated and quantitatively characterized design factor rather than as an optional add-on. Existing virtual piano research has already shown that users score significantly higher in clarity, realism, enjoyment, and satisfaction when kinesthetic and vibration feedback are added compared to no-feedback conditions. Studies that combine real-instrument baselines, experimental manipulation of haptic feedback, and longitudinal tracking would provide particularly strong tests of whether cross-modal compensation supports transferable musical skill rather than adaptation to a virtual interface. Is cross-modal compensation actually training musical skills, or is it merely training adaptation to a virtual interface (Fu et al., 2025; Pinkl et al., 2024)?
